# Understanding the influence of religious and safety concerns on childhood measles and pertussis vaccination: a study conducted in Aceh, Indonesia, 2022

**DOI:** 10.1186/s12879-025-11448-7

**Published:** 2025-09-26

**Authors:** Annie A. Murphy, Rosaria Indah, Amanda Yufika, Ichsan Ichsan, Tita Menawati Liansyah, Harapan Harapan, Daniel Birchok, Jason M. Pogue, Fitdha Khairadini, Abram L. Wagner

**Affiliations:** 1 Department of Epidemiology, School of Public Health, University of Michigan, Ann Arbor, 1415 Washington Heights, Ann Arbor, MI 48109 USA; 2 Medical Education Unit, School of Medicine, Universitas Syiah Kuala, Banda Aceh, Indonesia; 3 Department of Family Medicine, School of Medicine, Universitas Syiah Kuala, Banda Aceh, Indonesia; 4 Center for Global Health Equity, University of Michigan, Michigan Ann Arbor, United States; 5 Medical Research Unit, School of Medicine, Universitas Syiah Kuala, Banda Aceh, Indonesia; 6 Department of Microbiology, School of Medicine, Universitas Syiah Kuala, Banda Aceh, Indonesia; 7 Tsunami & Disaster Mitigation Research Center (TDMRC), Universitas Syiah Kuala, Banda Aceh, Indonesia; 8 Department of Pediatrics, School of Medicine, Universitas Syiah Kuala, Banda Aceh, Indonesia; 9 Tropical Disease Centre, School of Medicine, Universitas Syiah Kuala, Banda Aceh, Indonesia; 10 Department of Anthropology, Literature Science and the Arts, University of Michigan, Flint, MI USA; 11 Department of Clinical Pharmacy, College of Pharmacy, University of Michigan, Ann Arbor, MI USA

**Keywords:** Vaccine hesitancy, Vaccine barriers, Vaccine concerns, Allergies, Religion, Vaccine uptake, Aceh, Indonesia

## Abstract

**Background:**

Aceh, the westernmost province in Indonesia, was granted autonomous status, allowing the area to officially practice Sharia law, making the region religiously conservative. The province has the lowest measles vaccination rates in the country, with only 54% coverage. This study aims to quantify the contribution of concerns or structural barriers on non-vaccination.

**Methods:**

In a 2022 cross-sectional population-based study, 899 parents reported religious vaccine concerns, concerns about side effects, and whether they encountered structural barriers. The degree to which religious concerns impact the first dose of measles-rubella (MR1) and pentavalent (Penta1) vaccination was quantified through population-attributable fractions (PAF).

**Results:**

Among respondents, 62% reported their child had received MR1 and 63% Penta1. In total, 60% of parents expressed religious concerns about vaccination and 75% about vaccine side effects. The PAF for MR1 non-vaccination was 36% (95% CI: 21%, 52%) for religious concerns and 35% (95% CI: 16%, 51%) for concerns about side effects. For Penta1, the PAF was 42% (95% CI: 24%, 59%) for concerns about side effects and 28% (95% CI: 13%, 44%) for religious concerns. Structural barriers like stockouts, cost, or inconvenient clinic hours were less frequently cited and contributed minimally to non-vaccination.

**Conclusions:**

While concerns can overlap, evidence suggests that religious concerns are distinct and influence vaccination rates more than structural barriers. Understanding barriers to vaccination is a precursor to developing tailored interventions, like encouraging religious leaders to act as a trusted source of vaccine guidelines or promoting the use of a halal vaccine, that can mitigate hesitancy.

**Supplementary Information:**

The online version contains supplementary material available at 10.1186/s12879-025-11448-7.

## Introduction

Vaccination is a cornerstone of global public health, crucial for preventing infectious diseases and reducing mortality. However, vaccine hesitancy is a top-10 threat to global health, highlighting countries'ongoing challenges in increasing vaccination rates, particularly within marginalized communities [1, 2]. This global issue manifests in diverse ways across different countries and within regions of the same nations. Although Indonesia maintains a national vaccination coverage of 95.3%, coverage remains particularly low in Aceh, a province characterized by its religious conservatism and practice of Sharia law [3]. Within this province, vaccine hesitancy has contributed to persistently low immunization rates. In 2023, for instance, only 20.8% of Acehnese children were fully immunized [3]. The lack of high vaccination coverage leaves the province vulnerable to recurrent outbreaks of two of the most highly contagious yet vaccine-preventable diseases: measles and pertussis [3]. In 2022, Aceh reported the largest number of measles cases (978) compared to any province in Indonesia (4845 total) [4]. Aceh also has periodic pertussis outbreaks [5].

Vaccine hesitancy in this province is a complex issue influenced by diverse factors, including individual experiences and vaccine type. For example, insights from the local research team suggest that concerns surrounding measles vaccination are closely related to religion, whereas hesitations about the pertussis or pentavalent vaccination *may* stem from concerns about potential side effects. As Aceh remains a relatively understudied region of Indonesia, these insights underscore the need for expanded research to better understand the vaccine hesitancy dynamics and implement interventions.

Measles is highly contagious and can result in severe health complications, including death and adequate vaccination coverage can control its spread [6]. There has been a worldwide and sustained public health effort to eradicate measles, which would require around 95% vaccination coverage [7]. A 2017 study found that Aceh, reported only 54% measles vaccination coverage, allowing the virus to spread and contributing to a high rate of measles-related deaths in children under five (3.26 per 100,000 in 2019) [1, 8]. In 2019, the World Health Organization (WHO) of Southeast Asia set a goal to eliminate measles and rubella by 2023 [9]. Despite this goal, in 2022, Indonesia reported 2845 cases of measles, with Aceh accounting for 978 cases, making it one of the provinces with the highest incidence [4]. High vaccination coverage is essential for measles control, as demonstrated in Bangladesh, a majority Muslim country like Indonesia [10]. Bangladesh has had greater than 80% measles vaccination coverage since 2004, resulting in just 281 reported cases in 2022 [8]. The stark contrast between measles cases in Indonesia and Bangladesh highlights the effectiveness of immunization against this contagious disease and emphasizes the importance of vaccine research in Aceh.

Concerns surrounding the measles-rubella (MR) vaccine are often attributed to apprehensions about the halal status of the immunization [11]. In recent years, the Indonesian Council of Ulama (Majelis Ulama Indonesia or MUI) has lacked clear guidelines regarding its approval of the MR vaccine, leading to a significant drop in vaccination rates [1, 12, 13]. For example, the vaccine was labeled as haram, or forbidden under Islamic law, but later permitted by the MUI due to the lack of halal alternatives [1, 12, 13]. This uncertainty has resulted in several regions, including Aceh, reporting less than 10% new MR vaccination coverage [12].

In addition to measles, pertussis, commonly known as whooping cough, is preventable through the pentavalent (diphtheria, tetanus, pertussis, hepatitis B, Haemophilus influenzae type b; Penta) vaccine [14]. In 2022, Indonesia reported 414 cases, with a national Penta vaccination coverage of 85% [14]. However, regional disparities in vaccination coverage exist. For example, Aceh reported a 59.2% Penta vaccine coverage in 2019, leaving many individuals vulnerable to infection [15]. Since 1990, whooping cough has remained the sixth leading cause of death among children less than five years old in Aceh, accounting for 30.17 deaths per 100,000 live births in 2019 [8].

Concerns surrounding whooping cough or whole-cell pertussis, have been historically related to adverse reactions and side effects [11]. Side effects include redness, swelling, and fever, which occur in 1 in 100 vaccinations [11]. Other adverse outcomes, such as fever-induced seizures and hypotonic-hyporesponsive reactions, are less common [11]. These adverse outcomes, reported in the 1970 s and 1980 s, initiated a shift from whole-cell (DTwP) to acellular vaccinations (DTaP) in high-income countries [12]. However, due to the increased cost and limited global production capacity of DTaP, many countries, such as Indonesia, continue to use whole-cell DTP vaccination (within the Penta vaccine), leading to concerns about potential side effects and severe reactions [12, 13]. It remains unclear if these concerns are related to the low vaccination rates in Aceh, indicating the need for increased research in this area.

Recognizing that vaccine concerns are diverse and may vary by vaccine type, such as religious objections to the measles-rubella (MR) vaccine versus safety concerns about the whole-cell pertussis (DTwP) vaccine, we sought to better understand how these concerns influence vaccination behavior in Aceh. TABRIE, meaning “we deliver” in Acehnese, is an ongoing study and community project aimed at increasing vaccination coverage through community engagement strategies. As a first step, we conducted a baseline survey to assess factors contributing to vaccine hesitancy. The primary goal of this baseline study is to quantify the contribution of different types of concerns (religious objections, fears about side effects, and doubts about vaccine effectiveness) as well as structural barriers (distance, time, and cost) to childhood vaccination uptake. Using population attributable fractions (PAFs), we estimate the extent to which each barrier contributes to under-vaccination. Identifying the most influential concerns can help tailor future interventions to address the specific drivers of vaccine noncompliance in this context.

## Methods

### Study population

We conducted a multi-stage stratified cluster sampling approach in Aceh, the westernmost province on Sumatra Island in Indonesia. This was a baseline cross-sectional study to examine participants’ concerns about vaccination at a single point in time.

The study was conducted in Banda Aceh, the provincial capital with a population of 255,029 in 2021 [16], and Aceh Besar, the surrounding suburban district with a population of 409,527 [17]. These two locations were chosen because they represent an urban and suburban area in the province with the lowest vaccination coverage in Indonesia, and therefore serve as a starting point for understanding vaccination coverage in the province.

Banda Aceh comprises 9 subdistricts, while Aceh Besar consists of 23 subdistricts. A total of 14 subdistricts were randomly selected – 7 from each district. Household recommendations were obtained from community leaders based on the presence of eligible children. Enumerators visited villages within each selected subdistrict and conducted face-to-face interviews with parents or caregivers. Subsequent households were selected based on a list provided by the community leaders.

The survey aimed to generate estimates of Aceh’s vaccination coverage. We assumed this to be around 50%, a conservative assumption also based on our prior research [1]. Using an alpha of 0.05, and a power of 80%, we would need a sample of 383 to estimate the outcomes with a margin of error of 5%. Our best guess of a design effect of 2 came from our previous experiences. These yield an approximate sample size of 800.

Individuals were included in this study if they met the following inclusion criteria: (1) aged 18 years or older; (2) parent or guardian of a child 1–5 years old.

The study team is composed of researchers based in the US and Indonesia and includes several researchers fluent in both English and Bahasa Indonesia. The study team developed questions based on previously validated questions from the US CDC [18], and we added similarly worded questions related to religious concerns. The study team developed the questionnaire in hopes of gaining a better understanding of specific individual-level concerns and their potential impact on vaccination coverage. This study focused on identifying and contextualizing vaccine-based concerns to inform the development of future interventions, rather than repeating an existing health behavior theory.

The study team reviewed the questionnaire in English, translated it into Bahasa Indonesia, pre-tested the translation and made minor revisions, and then back-translated it into English. The questionnaire was administered in Bahasa, Indonesian. The questionnaire, in English and Bahasa Indonesian, is available at: 10.6084/m9.figshare.28352057.

The data collection team, including enumerators, comprised research staff and graduate students from Universitas Syiah Kuala. They received several hours of training, including mock interviews, prior to entering the field.

This questionnaire was conducted through participants’ completion of a written, printed survey. After the survey was completed, the study enumerators inputted the responses into Qualtrics, a software used to conduct surveys.

### Measurement of vaccination

During the interview process, we asked parents to provide us with their child’s vaccination card. If the parent had this card, we recorded information about the vaccination from this card. For parents without cards, we asked them to recall their child’s vaccination status. This approach is in line with other studies, including the Demographic and Health Survey program [19]. Due to communicability, vaccine preventability, and past vaccine campaigns, this study focuses on the administration of two vaccines: measles-rubella (MR) and pentavalent (diphtheria, tetanus, pertussis, hepatitis B, *Haemophilus influenzae* type b; Penta) vaccine. Information was collected about additional vaccines and can be seen in Table 3.

### Vaccination barriers and concerns

We asked participants various questions about vaccination concerns and structural barriers, with “Yes” and “No” as response options. Examples include: “The distance from my residence to the vaccination site is too far”, “Too costly”, “My children are allergic to vaccines”, “I am concerned about side effects from vaccines”, “I don’t think vaccines work very well”, “I am concerned that vaccines are not consistent with my religion (for example: that vaccines are not halal)”. All concerns were based on self-report and participants’ understanding of the question. For instance, the study team knew from prior experiences with the study population that individuals might overreport “allergies” (alergi in Bahasa Indonesia). Thus, this isn’t a purely medical issue but a social construct.

### Demographic covariates

We also asked participants their age, gender, ethnicity, and the highest level of education they have received.

### Statistical analysis

For our descriptive statistics, we reported 95% confidence intervals (CI) and used survey procedures that accounted for clustering of respondents within districts. We assessed the correlation between different concerns and barriers using Pearson’s correlation coefficient.

We tabulated the proportion who mentioned each barrier or concern, stratified by their vaccination status (having receipt of MR1 or not). We then calculated prevalence ratios (PR) using Poisson regression models with robust variance estimates [20]. This model’s outcome was no receipt of MR1, with a barrier or concern as the main predictor. This last model was, a priori, adjusted for child’s age and gender and respondent’s age, gender, ethnicity, and education.

For our main analysis, we constructed PAFs [20]. For each PAF, the outcome was non-receipt of MR1, and we separately calculated the PAF for each barrier or concern. The PAF was calculated as p(PR − 1)/PR, where p is the proportion of those with a barrier/concern among nonvaccinated individuals, and PR is the prevalence ratio of nonvaccination between those with a barrier/concern vs those without.

All analyses were conducted in SAS version 9.4 (SAS Institute, Cary, NC). We specified an alpha of 0.05, except for constructing the PAF, where the two inputs (the proportion and the prevalence ratio) had an alpha of 0.025 to mitigate multiple statistical inputs for each PAF.

To account for missing data or individuals opting out of answering a specific question, we did listwise deletion. If there was missing data, that individual was not included in the analysis for that variable.

We focus the main narrative text on the MR1 vaccine, but replicate the results with the Penta1 vaccine, shown in the appendix.

### Ethical approval

This study was approved by This study was approved by the Universitas Syiah Kuala ethical review board (#098/EA/FK/2023). The protocol was reviewed by the University of Michigan and exempted from their oversight (HUM00219083). All participants provided informed consent before any data collection.

## Results

Overall, we approached 1150 households within 19 sub-districts of Aceh province in Indonesia and 1096 (95.3%) households agreed to participate. These 1096 households had 2218 children < 18, but for this analysis, we have limited the sample to the 899 households that had a child 1–5 years of age, and further limited our analysis to the youngest child that the family had in this age range. Missing data, due to individuals opting out of answering a specific question, was mitigated through listwise deletion. Therefore, if there was missing data, that individual was not included in the analysis for that variable.

As seen in Table 1, of the questionnaire respondents, the majority were female (95%) due to the enumerators speaking to the individual who was home at the time of the survey. The high proportion of women respondents suggests that women are more likely than men to be home during the day. All but two respondents identified themselves as Muslims and most respondents (87%) identified as Acehnese.

**Table 1:** Distribution of demographic and socioeconomic characteristics of survey respondents, Aceh, Indonesia, 2022

	Overall Count	Column^a^ % (95% CI)	Count with MR1	% vaccinated with MR1 (95% CI)
Gender of child				
Male	456	51% (47%, 55%)	278	61% (54%, 68%)
Female	437	49% (45%, 53%)	279	64% (57%, 71%)
Missing	6		–	
Age of child				
1 year	211	23% (19%, 28%)	113	54% (42%, 65%)
2 years	256	28% (24%, 33%)	164	64% (56%, 73%)
3 years	191	21% (16%, 26%)	122	64% (56%, 72%)
4 years	165	18% (14%, 22%)	109	66% (56%, 76%)
5 years	76	8% (6%, 11%)	52	68% (57%, 80%)
Gender of respondent				
Male	47	5% (1%, 10%)	31	66% (48%, 84%)
Female	848	95% (90%, 99%)	526	62% (55%, 69%)
Missing	4		–	
Age of respondent				
18–29	358	40% (34%, 45%)	197	55% (43%, 67%)
30–39	469	52% (47%, 57%)	317	68% (62%, 73%)
40 +	69	8% (5%, 10%)	43	62% (52%, 73%)
Missing	3		–	
Monthly income of respondent				
< 5 million Rupiah (< $342)	584	93% (89%, 97%)	353	60% (51%, 70%)
≥ 5 million Rupiah (≥ $342)	45	7% (3%, 11%)	42	93% (85%, 100%)
Missing	270		165	61% (59%, 63%)
Religion of respondent				
Muslim	859	100% (99%, 100%)	544	63% (56%, 70%)
Catholic or Buddhist	2	0% (0%, 1%)	1	–
Missing	38			
Ethnicity of respondent				
Acehnese	783	87% (80%, 95%)	480	61% (55%, 68%)
Javanese	45	5% (2%, 8%)	30	67% (42%, 91%)
Other	68	8% (3%, 12%)	49	72% (59%, 85%)
Missing	3		–	
Highest level of education completed by respondent				
Less than elementary school or elementary school	51	6% (2%, 9%)	25	49% (33%, 65%)
Junior high school or Senior high school	414	46% (38%, 55%)	238	57% (52%, 63%)
Three years diploma, Bachelor’s degree, or Postgraduate degree	431	48% (38%, 58%)	295	68% (59%, 78%)
Missing	3		–	

^a^Percentage among those with a non-missing response

Concerns surrounding vaccine side effects were the most prevalent, with three-quarters (75%) of participants expressing their hesitancy as it relates to side effects (Table 2). Over half of the participants indicated concerns surrounding children’s allergies to the vaccine (63%), the vaccine’s consistency with religion (60%), and children’s fear of needles (60%). However, the majority of participants reported believing vaccines were safe for their children (54%) and effective at preventing illness (50%). Few participants reported structural barriers to vaccination, such as vaccine stockouts (11%), distance to the vaccine clinic (7%), the cost of the vaccine (7%), time off work (6%), and timing of clinics being inconvenient (6%).

**Table 2:** Attitudes, concerns, and barriers towards vaccination in Aceh, Indonesia, 2022

	Count	Percentage
Respondent is concerned about vaccine side effects		
Yes	673	75% (69%, 81%)
No	222	25% (19%, 31%)
Missing	4	
Respondent reports child allergic to the vaccine		
Yes	565	63% (53%, 74%)
No	330	37% (26%, 47%)
Missing	4	
Religious concerns		
Yes	539	60% (46%, 75%)
No	355	40% (25%, 54%)
Missing	5	
Respondent reports that child is scared of needles		
Yes	537	60% (50%, 70%)
No	359	40% (30%, 50%)
Missing	3	
Respondent is concerned that vaccines will be mandated through repressive forces		
Yes	491	55% (40%, 70%)
No	404	45% (30%, 60%)
Missing	4	
Respondent trusts that vaccines are safe for their children		
Strongly disagree	40	4% (0%, 9%)
Disagree	127	14% (8%, 20%)
Neither agree nor disagree	247	27% (15%, 40%)
Agree	369	41% (32%, 50%)
Strongly agree	116	13% (4%, 22%)
Respondent believes vaccines are effective at preventing illness		
Strongly disagree	36	4% (0%, 8%)
Disagree	134	15% (8%, 22%)
Neither agree nor disagree	269	30% (16%, 44%)
Agree	342	38% (29%, 47%)
Strongly agree	110	12% (3%, 21%)
Missing	8	
Respondent does not think vaccines are efficacious		
Yes	367	41% ((11)32%, 51%)
No	519	59% (49%, 68%)
Missing	13	
Vaccine stock-outs		
Yes	101	11% (2%, 21%)
No	790	89% (79%, 98%)
Missing	8	
Distance to clinic		
Yes	67	7% (4%, 11%)
No	828	93% (89%, 96%)
Missing	4	
Cost of vaccine		
Yes	61	7% (2%, 12%)
No	827	93% (88%, 98%)
Missing	11	
Time off work		
Yes	53	6% (2%, 10%)
No	839	94% (90%, 98%)
Missing	7	
Timing of clinics inconvenient		
Yes	52	6% (3%, 9%)
No	836	94% (91%, 97%)
Missing	11	

Among the barriers and concerns (Supplemental Table 1 in Additional Files 1), there were moderate correlations between time off work and the timing of clinics being inconvenient (*r* = 0.64) and concerns about side effects and children being allergic to the vaccine (*r* = 0.47). There were other weak correlations between distance to the clinic and time off work (*r* = 0.27), concerns about stock-outs and vaccine effectiveness (*r* = 0.24), and concerns about side effects and effectiveness (*r* = 0.24). We observed weak correlations between a child’s allergy to the vaccine and vaccine effectiveness (*r* = 0.25), and children being scared of needles (*r* = 0.25). Additionally, there were weak correlations between religious concerns and side effects (*r* = 0.29), vaccine effectiveness (*r* = 0.29), and a child’s allergy to the vaccine (*r* = 0.29). Religious concerns and perceptions of vaccine effectiveness in Aceh appear to function as independent belief systems, meaning that an individual's hesitancy due to religious reasons does not strongly predict their doubts about vaccine efficacy, and vice versa.

Of the 899 respondents, 62% reported their child having received one dose of the MR1 vaccine, and 63% with Penta1 (Table 3).

**Table 3:** Uptake of different vaccines, Aceh, Indonesia, 2022, *N* = 899

Vaccine^a^	Recommended age(s) at administration	Count vaccinated with 1 dose	Percentage vaccinated with 1 dose (95% CI)
MR vaccine	9, 18 months	560	62% (56%, 69%)
BCG vaccine	Birth dose	702	78% (73%, 83%)
HepB^b^ vaccine	Birth dose	734	82% (75%, 88%)
Penta^b^ vaccine	2, 3, 4, 18 months	570	63% (56%, 71%)
PCV vaccine	2, 3, 12 months	214	24% (14%, 34%)
OPV vaccine	1, 2, 3, 4 months	599	67% (58%, 75%)

*CI* confidence interval, *MR* Measles and Rubella, *BCG* Bacillus Calmette-Guerin, *HepB* Hepatitis B, *Penta* Pentavalent, *PCV* Pneumococcal Conjugate Vaccine, *OPV* Oral Polio Vaccine

^a^More dosing information available elsewhere (11)

^b^Pentavalent DTP-HepB-Hib vaccine available at 2, 3, 4, and 18 months

In terms of starting the MR vaccination series by social demographics (Table 1), of participants with a monthly income of less than 5 million Rupiah ($342), 60% of children received MR1. On the contrary, of participants with a monthly income greater than or equal to 5 million Rupiah ($342), 93% of children received MR1. Children’s MR1 vaccination rates were reported to be the lowest among parental respondents with less than elementary school or elementary school education (49%), followed by participants with a junior high school or senior high school level education (57%), and highest among participants with a three-year diploma, a Bachelor’s degree, or a Postgraduate degree (68%).

Table 4 shows the population-attributable fraction (PAF) of different vaccine-based concerns and barriers to not receiving MR1. Religious concerns, vaccine effectiveness, and side effects had similar PAFs, ranging from 35–36%, with overlapping confidence intervals. The PAF represents the proportional decline in non-vaccination that would occur if an intervention successfully mitigated all concerns. For example, a PAF of 36% associated with religious concerns indicates that MR1 non-vaccination would decrease from 38% by 36% to 24%, leading to an increase in MR1 non-vaccination from 62 to 76%. The PAF of children’s allergies as a barrier to MR1 vaccination (PAF: 21%, 95% CI: 7%, 36%) was lower than the aforementioned variables but higher than the PAFs for structural barriers to MR1 vaccination, which were negligible in size.

**Table 4:** PAF of barriers and concerns on not receiving a measles-rubella vaccine dose 1 (MR1), Aceh, Indonesia, 2022

	Proportion mentioning barrier/concern among those with MR1	Proportion mentioning barrier/concern among those without MR1	Prevalence ratio of not receiving MR1, in those with vs without barrier/concern ^a^	PAF	PAF Confidence Interval
Distance to clinic	6%	10%	1.42	3%	(0%, 7%)
Time off work	6%	6%	1.01	0%	(0%, 4%)
Timing of clinics inconvenient	6%	6%	1.24	1%	(0%, 6%)
Cost of vaccines	6%	8%	1.12	1%	(0%, 6%)
Vaccine stock-outs	10%	13%	1.16	2%	(0%, 9%)
Concerns about side effects	69%	85%	1.69	35%	(16%, 51%)
Concerns about vaccine effectiveness	29%	62%	2.21	34%	(23%, 46%)
Children scared of needles	57%	64%	1.18	10%	(−2%, 24%)
Children allergic to vaccine	58%	71%	1.41	21%	(7%, 36%)
Religious concerns	51%	75%	1.93	36%	(21%, 52%)

*PAF* population attributable fraction

^a^model adjusted for child’s age and gender and respondent’s age, gender, ethnicity, and educa

We include the results of Penta1 in Supplementary Tables 2–3 in Additional Files 1; patterns were similar to those for MR1. As expected, the PAF for Penta1 vaccination was the largest for concerns about side effects (PAF: 42%, 95% CI: 24%, 59%).

## Discussion

This baseline questionnaire from the TABRIE project analyzed the contribution of structural barriers, personal concerns, and vaccine hesitancy on childhood vaccination status, specifically in regard to measles-rubella dose 1 (MR1) and pentavalent dose 1 (Penta1). These vaccines were specifically selected based on the study team’s insights that concerns surrounding measles vaccination appear to be closely related to religion, whereas hesitations about the pertussis or pentavalent vaccination may stem more from concerns about potential side effects. This paper specifically focuses on a cross-sectional analysis of in-person surveys collected from 899 households in Aceh, Indonesia, a predominantly Muslim and religiously conservative area [1]. Overall, the results of the survey indicated that personal concerns such as allergies, vaccine effectiveness, vaccine safety, and religious concerns contributed to high rates of vaccine hesitancy. Interestingly, logistical and physical barriers, such as distance to vaccination clinics and time off work, did not heavily influence childhood vaccination status with MR1 or Penta1.

We note in our study that evaluated several concerns and structural barriers. Many of the concerns were significantly, but weakly, correlated. The weak correlations suggest that, although there is some overlap, religious concerns and other vaccine-related concerns largely operate independently rather than mutually reinforcing each other. However, an indirect relationship may persist. For example, religious leaders who express skepticism about vaccines may unintentionally influence both perceptions of religious permissibility and beliefs about scientific effectiveness. This underscores the nuanced nature of vaccine hesitancy and the need for interventions that address religious objections and vaccine misinformation as distinct yet potentially interconnected challenges [21]. Research in this area is on-going but there are some suggestive results from experiences in the United States during the COVID-19 pandemic. For instance, faith leaders within minority communities in the United States are often well-respected and trusted, which enables them to correct vaccine misinformation, particularly in relation to religious matters [21].

### Vaccination and religion

One of the most prominent impacts we saw on childhood vaccine non-compliance was religious concerns. Almost all participants in the TABRIE project were Muslim, and roughly half of the respondents noted religious concerns as a barrier to childhood vaccination. Halal is a certification awarded to food that is deemed “lawful” under the Quran and issued by the Majelis Ulama Indonesia (MUI), an institution that authorizes halal certification for food products [22, 23]. When an item is labeled as halal, it has been verified to be free of pork and other contaminants at all levels of the creation and distribution [22]. For many Muslims, the significance of halal status extends past the exclusion of prohibited items and signifies food that is “pure, sacred, appropriate or healthy” [22]. These religious values expand beyond food items and directly influence the perception of vaccines. In terms of childhood vaccinations, many studies have reported findings surrounding concerns over vaccines being haram, or something that is prohibited by Islamic law, due to the inclusion of pork in the manufacturing of the vaccines [24]. Studies have also found that parents refuse childhood vaccinations due to the concern of vaccines destroying healthy cells [24]. Excluding the new MR vaccine, which the MUI has *permitted*, immunizations have been approved and encouraged through halal verification [24]. However, many individuals continue to cite religious concerns as a barrier to vaccination due to their distrust of governmental influence on the MUI [24]. Because of the belief that a halal diet confers a healthy lifestyle, it is possible that religiously conservative individuals deny childhood vaccinations as they presume natural immunity to be sufficient to fight off pathogens and infections [24]. To increase vaccine uptake in Aceh and other provinces in Indonesia, public health officials should focus on establishing trust in the vaccine halal certifications and effectively communicating them at the individual and community levels. For example, a study conducted in Yogyakarta Province, Indonesia, explored the role of religious leaders in vaccine promotion through qualitative interviews [25]. The findings indicated that religious leaders saw the potential for them to act as intermediaries between vaccine production and religious acceptance [25]. They advocated for closer involvement in the MUI fatwa process to support the endorsement of vaccines [25]. Additionally, religious leaders expressed interest in working with pharmaceutical companies to gain a clearer understanding of vaccine manufacturing, particularly regarding information about ingredients and the purification of porcine products [25]. Religious leaders suggested that such knowledge would enable them to more effectively promote vaccines to their congregations and give them the ability to respond to questions and concerns with greater confidence and accuracy, thereby increasing the trust that individuals have in the vaccine’s safety, religious alignment, and efficacy [25].

Religious conservatism, especially in Muslim majority countries, is often associated with low vaccination rates [26]. Besides Indonesia, other Muslim majority countries, like Pakistan and Malaysia, struggle with religious fatalism and some religious leaders discouraging the use of the vaccine [27]. However, other Muslim-majority countries in Africa have promoted vaccination. For example, the Dakar Declaration of Vaccines, issued by a group of African Muslim scholars and medical professionals, cites texts from the Qur’an and addresses concerns about vaccines while promoting vaccination [28].

### Vaccination and concerns about side effects and allergies

Perceptions about vaccine safety have been extensively studied, particularly in the context of the Health Belief Model (HBM). This model suggests that when making a health-related decision, individuals weigh the susceptibility, severity, safety, efficacy, and social pressures before engaging in the behavior [29]. The HBM has been used in an international context with studies including participants from Malaysia, Indonesia, and India [30]. Given that safety and efficacy are key pillars of health decision-making, it is unsurprising that concerns about vaccine safety and side effects were prominent in our study. For example, concerns about side effects were particularly high in relation to the Penta1 vaccine, likely due to the high rates of side effects reported with the use of whole cell pertussis vaccines (DTwP) included in the vaccine [11]. While many high-income countries have shifted to acellular pertussis vaccines (DTaP) to decrease the prevalence of side effects associated with the immunization, many countries, like Indonesia, continue to use the DTwP due to cost and supply restraints [12, 13].

In the same vein of safety concerns, this study found that concerns about allergies were another impediment to not receiving a vaccine. There are several interpretations of “allergy”, each with various levels of medical severity, ranging from an upset stomach and headache to a reaction requiring medical attention. While the TABRIE survey question related to allergies was broad and did not limit the term “allergy” to a medical contraindication, over half of the respondents’ noted allergies as a barrier or concern for vaccinating their children. This rate is much higher than prevalence of medical contraindications or allergies to routine vaccinations seen in other studies, for example 1.06% in a study conducted Hangzhou, China [31]. There are several explanations for the extremely abnormal and high reported allergy rate observed in the study participants. First, “allergy” can be perceived as any discomfort or sickness, emphasizing that individuals may have reported an allergy as a barrier to vaccination due to the uncomfortable sensation of receiving an immunization. Secondly, since the rate of allergy concerns in our study was well above the expected rate of medical contraindications to vaccines, we suggest that individuals may be using these concerns as an excuse to avoid vaccinating their children. For example, in the Hangzhou study, 13.53% of the medical contradictions were misdiagnosed by vaccination doctors [31]. It is possible that the observed rate of concerns surrounding vaccine allergies in Aceh, Indonesia, is due to false positives or false identifications. In California, researchers observed a 250% increase in medical exemptions, including allergies, to vaccines one year after the passage of a law (SB277) that removed nonmedical exemptions as acceptable reasons for childhood non-vaccination before entering school [29]. The sharp and sudden increase in medical exemptions demonstrates that individuals in California may be using allergies or minor medical exemptions as a means of avoiding vaccine mandates [29]. Moreover, misinformation about allergies, side effects, or long lasting health effects may contribute to the high rates of reported vaccine allergies, as a similar phenomenon has been reported in the United States with regard to COVID-19 vaccine hesitancy [32]. To combat these concerns, public health officials in Aceh should focus on accurate diagnoses and the dissemination of accurate information about rare vaccine allergies at the Puskesmas, through the media, or communicated by religious leaders, as they are often viewed as trusted members of society [25].

### Vaccination and structural barriers

Access and affordability of vaccines are often credited with having a strong influence on non-vaccination rates [33]. Factors such as convenience, timing, location, and finances fall into these categories [33]. However, our preliminary survey found that logistical barriers were less significant than concerns surrounding vaccine safety, efficacy, and religion, suggesting the need to focus interventions on awareness and uptake in Aceh, Indonesia.

### Public health implications

Our study findings are limited to the concerns expressed by parents and their relationship to non-vaccination. Our findings indicate that some concerns, particularly as they relate to religion and perceptions about allergies and side effects, could be useful starting points for interventions across a number of different levels.

### Governmental health reform

While public health interventions often prioritize improving vaccine access and availability, our findings suggest that in Aceh, Indonesia, the strong healthcare infrastructure has minimized structural barriers and their impact on vaccine hesitancy. For example, the cities of Banda Aceh and Aceh Besar host over 130 community health centers (Puskesmas and Puskesmas Pembantu), enhancing the accessibility of public health services in the province. Given that the results of this study suggest that structural barriers, such as access and accessibility, are less influential in vaccine noncompliance than vaccine-related hesitancy (safety, religion, side effects, etc.), we recommend that public health officials shift their focus from improving access alone to developing interventions specifically aimed at reducing hesitancy. For example, health care workers and community health workers should receive training on *community-specific* concerns to effectively and accurately respond to alleviate fears.

Assuming there are limited structural barriers to vaccination, the existing literature explores how healthcare workers and community leaders can discuss vaccines, particularly with more skeptical parents. Much of the theoretical basis of this literature is from North America and Europe, but it emphasizes techniques such as using nudges and motivational interviewing [34]. Nudges can be used to increase immunization rates by presumptively vaccinating children during office visits [34]. This method requires individuals or parents to opt out of vaccination instead of opting in [34]. On the other hand, motivational interviewing involves reflective listening and open-ended questions that lead individuals to consider inconsistencies in their beliefs, rather than the provider directly trying to persuade the individual to change their beliefs [34]. The translation of these ideas into non-Western contexts is not clear; however, these innovative techniques may be an avenue for overcoming religious and safety concerns.

These suggested interventions align with the goals of Indonesia’s Integrated Primary Health Care Initiative, launched in 2022 [35]. This governmental reform aims to restructure primary health care delivery, providing a more streamlined approach to care and integrating services [35]. A key aspect of this initiative involves establishing local area monitoring systems to track the progress of initiatives and targets [35]. These monitoring systems can track trends across Puskesmas, enabling public health officials to identify and address areas that require attention [35]. Additionally, to promote standardized care, this initiative will implement enhanced training programs for health workers [35]. Although not currently included in the initiative’s plans, the implementation of training programs may provide public health officials the opportunity to equip providers and community health workers with specific skills required to address vaccine hesitancy (nudges, motivational interviewing). Therefore, there is a potential opportunity to connect the TABRIE project’s aims with Indonesia’s Primary Health Care Initiative, thus having a greater impact on the population’s health.

### Vaccine availability, including non-gelatin vaccines

To reduce halal apprehension related to vaccination, public health officials can lobby for widespread distribution of vaccine alternatives without gelatin, a common porcine ingredient. For instance, there is a gelatin-free Measles, Mumps, and Rubella vaccine called Priorix GSK-MMR, and its introduction into Indonesian vaccine schedules would remove religious concerns specific to the halal status of the vaccine [36]. This vaccine was approved by the United States Food and Drug Administration in 2022, but it may not yet be available on the global market [37].

### Religious leaders

Finally, we suggest that the use of religious organizations and leaders could garner trust surrounding vaccines. Several studies have found that faith-based organizations can help individuals overcome physical barriers to health behaviors, according to a systematic review incorporating studies from across the globe, including Southeast Asia [26]. In Indonesia specifically, vaccines can be further popularized in the religious setting through fatwas, rulings, and the incorporation of religious leaders as active participants in the development and dissemination of vaccine knowledge, as mentioned above [25]. Members often trust their religious leaders, highlighting the potential for leaders to make an impact when encouraging health-promoting behaviors [26]. In another religious context, during the COVID-19 pandemic, Black pastors in the US played a key role in promoting vaccination among the members of their churches [38]. The churches used their relationship with their community to spread information and build trust, acted as an administration site to reduce barriers, and pastors got vaccinated themselves to lead by example [38]. Similar tactics and techniques can be used by religious leaders in Indonesia to increase the uptake of routine childhood vaccinations.

### Future directions

As the TABRIE research study progresses, we aim to explore how socioeconomic factors mediate personal concerns and vaccination decisions. In particular, we seek to better understand how variables, such as income and education, contribute to vaccine noncompliance. Additionally, we believe it would be helpful for future research to disentangle concerns related to vaccine side effects (75%) and vaccine safety concerns (54%). This disconnect may highlight a wording issue within the questionnaire or showcase the long-standing impact and discussion surrounding the side effects associated with the whole-cell pertussis vaccine. Finally, we hope to examine the influence of sociocultural characteristics and Acehnese governmental influence on vaccination decisions. By broadening the focus areas, we hope to better understand the reasons underlying vaccine hesitancy in Aceh and develop tailored interventions.

### Strengths and limitations

This study has several strengths. This study used population-based surveys created from validated US CDC questionnaires as the basis [18]. Aceh is a relatively understudied.

area of Indonesia, with significantly high rates of non-vaccination, highlighting the importance of increased research in this area. Additionally, Aceh is an important study setting due to its religious conservatism. Religious affiliation has been associated with low vaccination coverage, especially in Muslim majority nations, emphasizing the need for research and tailored interventions in these areas [27]. Additionally, through collaboration between the University of Michigan and Universitas Syiah Kuala, the research team was able to expand an existing relationship with community members, resulting in a very high participation rate (95%).

This study also has several limitations. The cross-sectional nature of this study limits the ability to establish temporality and prevents researchers from following up with participants to gather additional information after data collection. However, the TABRIE project is multi-step, therefore allowing the opportunity to further explore vaccine concerns in the Aceh community. While this study may have been subject to selection bias due to the possibility that individuals with more positive vaccine attitudes were more likely to agree to participate, random selection of subdistricts lessened the bias’s impact on the results. Due to this study’s quantitative methods, we were unable to fully explore individuals’ reasons for vaccine concerns. To mitigate this, we utilized the study's results and previous literature to provide a possible explanation for the most prevalent concerns. Vaccine hesitancy is a multifaceted issue, thereby resulting in a potential correlation between vaccine concerns, resulting in similar PAF values and entangled results. To reduce the impact of overlapping concerns and on our interpretations and increase transparency, we created a correlation table, which can be found in the Supplementary files. Additionally, participants may not have answered questions completely or stated “Prefer not to say” or “Don’t know” due to social desirability bias and hope to provide socially “acceptable” answers. To combat missing data, we performed listwise deletion, which excluded individual-level missing data from variable analysis. Finally, we acknowledge that there could have been differences in participants who gave us access to vaccination card information vs those who did not. While this is a clear limitation, the research team gave all participants the opportunity to present a vaccine card.

## Conclusion

In a pilot study of the TABRIE project, we assessed how structural barriers, personal concerns, and religious beliefs influence uptake of the first doses of measles-rubella and pentavalent vaccines in Aceh, Indonesia. Our findings suggest that non-structural concerns, particularly fears about vaccine side effects and religious concerns, are prominent contributors to under-vaccination. In contrast to other settings where logistical barriers such as distance to clinics or time off work are major obstacles, we found that such structural factors played a minimal role in this context. This underscores the need for interventions that go beyond improving access and instead focus on building trust in vaccines. Future work should explore the potential of engaging religious leaders as trusted messengers, develop targeted health education campaigns, and incorporate community-driven approaches to address specific fears and misconceptions about vaccines.

## Supplementary Information

Supplementary Material 1

## Data Availability

The questionnaire used and data collected are available at: 10.6084/m9.figshare.28352057.

## Abbreviations

WHO: World Health Organization
MR: Measles-rubella vaccine
MR1: First dose of the measles-rubella vaccine
Penta: Pentavalent vaccine
Penta1: First dose of pentavalent vaccine
PAF: Population-attributable fractions
MUI: Indonesian Council of Ulama or Majelis Ulama Indonesia
DTwP: Diphtheria, Tetanus, and Whole-Cell Pertussis vaccine
DTaP/DTP: Diphtheria, Tetanus, and Pertussis vaccine
