# Supplementary material for: Understanding the influence of religious and safety concerns on childhood measles and pertussis vaccination: a study conducted in Aceh, Indonesia, 2022
Source: BMC Infect Dis. 2025 Sep 26;25:1140. doi: 10.1186/s12879-025-11448-7 (PMC12465582; doi:10.1186/s12879-025-11448-7)
Supplement: Supplementary file 1 — Supplementary Material 1 [file 12879_2025_11448_MOESM1_ESM.docx]

**Supplemental Table 1.** Correlation between concerns and barriers, Aceh, Indonesia, 2022.

|  | 1 | 2 | 3 | 4 | 5 | 6 | 7 | 8 | 9 | 10 |
| --- | --- | --- | --- | --- | --- | --- | --- | --- | --- | --- |
| 1. Distance to clinic | 1.00 |  |  |  |  |  |  |  |  |  |
| 2. Time off work | 0.27** | 1.00 |  |  |  |  |  |  |  |  |
| 3. Timing of clinics inconvenient | 0.15** | 0.64** | 1.00 |  |  |  |  |  |  |  |
| 4. Cost of vaccines | 0.18** | 0.05 | 0.01 | 1.00 |  |  |  |  |  |  |
| 5. Vaccine stock-outs | 0.07* | 0.09* | 0.12* | 0.05 | 1.00 |  |  |  |  |  |
| 6. Concerns about side effects | -0.02 | -0.05 | -0.03 | 0.10* | -0.03 | 1.00 |  |  |  |  |
| 7. Concerns about vaccine effectiveness | 0.04 | -0.04 | -0.005 | 0.17** | 0.24** | 0.24** | 1.00 |  |  |  |
| 8. Children scared of needles | 0.02 | 0.02 | 0.04 | 0.01 | 0.01 | 0.14** | 0.12* | 1.00 |  |  |
| 9. Children allergic to vaccine | -0.02 | 0.005 | 0.01 | 0.10* | -0.05 | 0.47** | 0.25** | 0.25** | 1.00 |  |
| 10. Religious concerns | 0.08* | -0.05 | -0.05 | 0.09* | 0.06 | 0.29** | 0.29** | -0.07* | 0.29** | 1.00 |

| <-0.40 | -0.39 to -0.30 | -0.29 to -0.20 | -0.19 to -0.10 | -0.09 to 0.09 | 0.10 to 0.19 | 0.20 to 0.29 | 0.30 to 0.39 | >0.40 |
| --- | --- | --- | --- | --- | --- | --- | --- | --- |

**Note:**

* P<0.05, ** P<0.0001

**Supplemental Table 2.** Vaccination status by demographic characteristics, Aceh, Indonesia, 2022.

|  | Child vaccinated with Penta1 | |
| --- | --- | --- |
|  | Count | Percent |
| Gender of child |  |  |
| Male | 279 | 61% (53%, 69%) |
| Female | 287 | 66% (57%, 75%) |
| Missing (n=6) | – |  |
| Child age (year) |  |  |
| 1 year | 129 | 61% (51%, 72%) |
| 2 years | 168 | 66% (56%, 75%) |
| 3 years | 120 | 63% (51%, 75%) |
| 4 years | 107 | 65% (53%, 77%) |
| 5 years | 46 | 61% (51%, 70%) |
| Gender of respondent |  |  |
| Male | 25 | 53% (35%, 72%) |
| Female | 541 | 64% (56%, 72%) |
| Missing (n = 4) | – |  |
| Age of respondent |  |  |
| 18-29 | 200 | 56% (44%, 68%) |
| 30-39 | 321 | 68% (61%, 76%) |
| 40+ | 47 | 68% (54%, 82%) |
| Missing (n = 3) | – |  |
| Income of respondent |  |  |
| < 5 million Rupiah (< $342) | 358 | 61% (52%, 71%) |
| ≥ 5 million Rupiah (≥ $342) | 38 | 84% (66%, 100%) |
| Missing (n = 270) | 174 | 64% (55%, 74%) |
| Religion of respondent |  |  |
| Muslim | 554 | 64% (57%, 72%) |
| Catholic or Buddhist | 0 | – |
| Missing | 38 |  |
| Ethnicity of respondent |  |  |
| Acehnese | 487 | 62% (55%, 70%) |
| Javanese | 30 | 67% (47%, 87%) |
| Other | 51 | 75% (60%, 90%) |
| Missing (n = 3) | – |  |
| Highest level of education completed by respondent |  |  |
| Less than elementary school or elementary school | 20 | 39% (13%, 66%) |
| Junior high school or Senior high school | 245 | 59% (52%, 66%) |
| Three years diploma, Bachelor’s degree, or Postgraduate degree | 302 | 70% (60%, 80%) |
| Missing (n = 3) | – |  |

**Supplemental Table 3.** Population attributable fraction of various barriers and concerns on not receiving pentavalent vaccine dose 1 (Penta1), Aceh, Indonesia, 2022.

|  | Proportion mentioning barrier / concern among those with Penta1 | Proportion mentioning barrier / concern among those without Penta1 | Prevalence ratio of not receiving Penta1, in those with vs without barrier / concern ^a^ | PAF | PAF Confidence Interval |
| --- | --- | --- | --- | --- | --- |
| Distance to clinic | 7% | 8% | 1.04 | 0% | (-1%, 4%) |
| Time off work | 6% | 5% | 0.91 | -1% | (-1, 3%) |
| Timing of clinics inconvenient | 4% | 8% | 1.56 | 3% | (0%, 7%) |
| Cost of vaccines | 5% | 9% | 1.30 | 2% | (0%, 8%) |
| Vaccine stock-outs | 11% | 12% | 1.02 | 0% | (0%, 6%) |
| Concerns about side effects | 69% | 87% | 1.96 | 42% | (24%, 59%) |
| Concerns about vaccine effectiveness | 28% | 64% | 2.44 | 38% | (26%, 50%) |
| Children scared of needles | 58% | 63% | 1.09 | 5% | (-6%, 20%) |
| Children allergic to vaccine | 57% | 73% | 1.57 | 27% | (12%, 43%) |
| Religious concerns | 54% | 72% | 1.64 | 28% | (13%, 44%) |

**Notes**

PAF, population attributable fraction

^a^ model adjusted for child’s age and gender and respondent’s age, gender, ethnicity, and education
